# Что нового принес эндокринологии ушедший 2022 год?

**DOI:** 10.14341/probl13261

**Published:** 2023-02-25

**Authors:** Г. А. Мельниченко, М. В. Шестакова

**Affiliations:** Национальный медицинский исследовательский центр эндокринологии; Национальный медицинский исследовательский центр эндокринологии

**Keywords:** сахарный диабет, ожирение, нейроэндокринные опухоли, тераностика, дислипидемия

## Abstract

В ушедшем году детально проработан и представлен на утверждение в Правительство РФ Федеральный проект «Борьба с сахарным диабетом» 2023–2030, который позволит существенно улучшить практику работы с больными сахарным диабетом, обеспечив им максимальную доступность медицинской помощи, в том числе в обновленных и технологически переоснащенных региональных эндокринологических центрах, возродив активную работу «Школ диабета», Кабинетов «диабетической стопы», диагностических лабораторий, внедряя новые формы коммуникации с пациентами, в том числе с использованием персональных помощников врача, непрерывных технологий мониторинга гликемии и др.

## Abstract (en)

In the past year, the Federal Project “Fight against Diabetes” 2023–2030 was developed in detail and submitted in detail and submitted to the Government of the Russian Federation, which will significantly improve the practice of working with patients with diabetes, providing them with maximum availability of medical care, including in updated and technologically re -equipped regional endocrinological centers, reviving the active work of “Diabetes schools”, “Diabetic foot” rooms, diagnostic laboratories, introducing new forms of communication with patients, including using personal assistants of a doctor, continuous technologies for monitoring glycemia, etc.

Начнем с самого главного — в ушедшем году детально проработан и представлен на утверждение в Правительство РФ Федеральный проект «Борьба с сахарным диабетом» 2023–2030, который, как мы все надеемся, позволит существенно улучшить практику работы с больными сахарным диабетом (СД), обеспечив им максимальную доступность медицинской помощи, в том числе в обновленных и технологически переоснащенных региональных эндокринологических центрах (РЭЦ), возродив активную работу «Школ диабета», Кабинетов «диабетической стопы», диагностических лабораторий, внедряя новые формы коммуникации с пациентами, в том числе с использованием персональных помощников врача, непрерывных технологий мониторинга гликемии и др. Федеральный закон Президента РФ В. Путина от 5 декабря 2022 г. N 466-ФЗ «О федеральном бюджете на 2023 год и на плановый период 2024 и 2025 годов» отдельной строкой утвердил финансирование мероприятий в рамках федерального проекта «Борьба с сахарным диабетом» на 2023–2025 гг.

Не самый простой в политическом и экономическом отношении год, тем не менее, стал годом активной работы Правительства, Министерства здравоохранения, Российской Ассоциации эндокринологов ради лиц с одним из самых социально значимых заболеваний — сахарным диабетом. Думаю, что в ближайших номерах журнала «Проблемы эндокринологии» мы прочтем еще много статей о прекрасной практической и научной деятельности в этом направлении. Сейчас важно, что новый Федеральный проект борьбы с сахарным диабетом позволяет поднять на более высокий организационный, технологический и методический уровни ту работу, которая так успешно проводилась в период 1996–2012 гг., когда работала Федеральная программа «Сахарный диабет», позволившая создать структурированную диабетологическую службу на всей территории РФ.

А теперь хотелось бы рассказать о мировых научных достижениях 2022 г. в разных областях эндокринологии.

## В ОБЛАСТИ САХАРНОГО ДИАБЕТА

17 ноября 2022 г. впервые в истории диабетологии FDA (США) зарегистрировало препарат — теплизумаб — для замедления развития СД 1-го типа (СД1) у взрослых и детей в возрасте 8 лет и старше, у которых в настоящее время диагностирован СД1 на «доклинической» стадии, т.е. при наличии дисгликемии недиабетического уровня и нескольких специфических антител к бета-клеткам поджелудочной железы. Одобрение препарата было ­получено по итогам исследования TriaL Net-10, в ­котором внутривенно вводили тепли­зумаб (­антитело к СD3) в течение 14 дней и получили снижение на 40% вероятности развития СД1 за период наблюдения [1]. При долгосрочном наблюдении за участниками исследования было зафиксировано, что клинический дебют СД1 удавалось отсрочить на 2,5–3 года! Несмотря на относительную скромность исследования, нельзя не выделить принципиально новый долгожданный результат: появилось антитело, способное существенно замедлить развитие СД1 у лиц с высокой вероятностью его развития (родственники лиц с СД1, имеющие антитела к глутаматдекарбоксилазе и дисгликемию недиабетического уровня). Надо сказать, что более ранние работы по применению этого же препарата у людей с впервые выявленным СД1 (но не на доклинической стадии его развития) оказались неэффективны, что лишний раз подчеркивает важность «терапевтического окна« времени вмешательства.

Арсенал средств для лечения СД 2 типа (СД2) пополнился новым препаратом — двойным агонистом рецепторов глюкагоноподобного пептида 1-го типа и глюкозозависимого инсулинотропного полипептида. Этот препарат — Тирзепатид (Mounjaro™) — был одобрен в мае 2022 г. FDA (США) для подкожных инъекций 1 раз в неделю для контроля гликемии. В программе исследований SURPASS (всего 8 исследований 3-й фазы) было показано, что препарат не только эффективно снижает уровень гликированного гемоглобина (HbA1c), но и превосходит все препараты сравнения по степени снижения массы тела. В США препарат одобрен в 3 дозах (5, 10 или 15 мг). Лечение препаратом приводит к снижению HbA1c на 1,8–2,0 пункта и массы тела на 7–10 кг (в зависимости от дозы) в течение 40 нед лечения. 2/3 участников исследования (более 60%), принимавших самую высокую дозу, удалось снизить массу тела на 20% [2]. Таким образом, на сегодняшний день этот препарат является непревзойденным по эффективности средством для лечения СД2 в сочетании с ожирением. В 2023 г. планируется ускоренное рассмотрение FDA нового показания для этого препарата, а именно лечения ожирения без сопутствующего СД2.

Радостное событие случилось в 2022 г. в жизни пациентов с СД2 и хронической болезнью почек (ХБП): в США и Европе зарегистрирован первый представитель нового класса препаратов — нестероидный антагонист минералокортикоидных рецепторов (МКР) финеренон, который в серии рандомизированных клинических исследований (FIDELIO-DKD и FIGARO-DKD, объединенных в программу FIDELITY) доказал высокую эффективность в качестве кардионефропротектора. По истечении 3 лет терапии у пациентов с СД2 и ХБП, получавших финеренон в добавление к максимально переносимым дозам ингибиторов ренин-ангиотензиновой системы, отмечалось значимое снижение рисков сердечно-сосудистых событий на 14% (Р=0,0018) и почечных событий на 23% (P=0,0002), независимо от исходных значений расчетной скорости клубочковой фильтрации, уровня альбуминурии, HbA1c [3]. Механизм нефропротективного действия препарата связывают с противовоспалительным и антифибротическим действиями антагонистов МКР на уровне почечного интерстиция, что является уникальным и не присущим другим классам нефропротекторов (блокаторам ренин-ангиотензиновой системы и глифлозинам). Существенное преимущество этого класса препаратов в отличие от стероидных антагонистов МКР — значимо меньшее развитие побочных явлений, таких как гиперкалиемия и гирсутизм [4]. В 2022 г. препарат финеренон был включен в клинические рекомендации США нефрологического и эндокринологического профиля для применения у пациентов с СД2 и ХБП с наивысшим уровнем доказательности «А» (KDIGO-2022, AACE-2022, ADA-2022). В России регистрация этого препарата ожидается весной 2023 г.

## В ОБЛАСТИ КЛИНИЧЕСКОЙ ЭНДОКРИНОЛОГИИ

После работы в условиях пандемии мы все стали свидетелями активного внедрения в нашу практику принципиально новых для эндокринолога лекарств, ранее входивших в основном в арсенал ревматологов, — антител к фрагментам клеток или клеточным рецепторам, а ­также селективных рецепторных модуляторов.

Важным оказался успех антитела к рецептору инсулиноподобного фактора роста 1-го типа, тоцилизумаба, в лечении эндокринной офтальмопатии и, как выяснилось впоследствии, претибиальной микседемы. R. Crespo-Trevino и соавт. [5] доказали эффективность препарата и при этом ассоциированном с болезнью Грейвса аутоиммунном поражении.

Вместе с тем уже ставшее привычным для наших врачей участие в многоцентровых исследованиях в изменившейся геополитической ситуации 2022 г. было прервано, и многие исследования, в которых планировалось участие врачей нашей страны, стали недоступными. Тем важнее те исследования, которые закончились в 2022 г., результаты которых в ближайшем будущем могут изменить нашу практику. В первую очередь это относится к препарату осилдростат (истуриза), ингибитору 11-бета-гидроксилазы, отличающемуся от разрешенного ЕМА и используемого в течение более 50 лет препарата метопирон (метирапон) большей продолжительностью действия и меньшим количеством побочных эффектов. В 2022 г. опубликованы результаты двух многоцентровых исследований [6][7], подтвердивших высокую эффективность препарата, позволившую на сегодняшний день рекомендовать этот ингибитор стероидогенеза ЕМА и FDA при лечении всех форм гиперкортизолизма, если не достигнуто излечение хирургическим путем.

Постоянно расширяется использование, особенно в сфере нейроэндокринологии и онкоэндокринологии, пептидов как носителей радиоактивной метки с двойным применением — тераностиков, то есть препаратов, используемых с диагностической и лечебной целью. Потенциальный переворот в диагностике кортикотропином, позволяющий, вероятно, уточнять и латерализацию, может достигаться при проведении ПЭТ/КТ с использованием меченного галлием кортиколиберина 68Ga CRH [8]. Из 24 наблюдений в 100% случаев была получена идентификация кортикотропином, причем даже не выявляемых на МРТ (!). Дополнительное наложение изображения ПЭТ на МРТ обеспечивало латерализацию опухоли, при этом в случаях эктопических опухолей, продуцирующих адренокортикотропный гормон (АКТГ), захвата в гипофизе не выявляли. Если дальнейшие исследования на большем количестве больных подтвердят эти данные, у нас появится надежная неинвазивная альтернатива катетеризации каменистых синусов, причем с лучшими результатами.

Накапливаются новые молекулы для лечения нейроэндокринных опухолей и опухолей гипофиза, и уже появляются пероральные формы — так, компания Сrinetics завершает уже третью фазу изучения агониста соматостатинового рецептора 2 типа (SSSR 2) палтусонида, представителя принципиально нового класса оральных селективных небелковых связывающихся с рецепторами молекул. Палтусонид — маленькая молекула, структурно ничего общего с соматостатином не имеющая, оказывает чисто конформационное влияние на рецептор. Предварительные итоги начатого в 2021 г. проспективного исследования крайне обнадеживают [9].

В настоящее время препарат находится в третьей фазе испытаний при лечении акромегалии и во второй фазе как препарат для лечения нейроэндокринных опухолей, в том числе карциноидов [10].

Разработан подобного же типа агонист рецепторов 5-го типа (ssr5), пока известно только кодовое название CRN04777, для лечения неонатального гиперинсулинизма.

Активно выходят на мировую арену препараты для лечения моногенных форм ожирения, начата IIb фаза изучения препарата Tesomet — фиксированной комбинации ингибитора обратного захвата моноаминов и β-1-селективного блокатора для лечения синдрома Прадера–Вилли [11]; при таких орфанных синдромах, характеризующихся гиперфагией, как синдромы Альстрема и Барде–Бидля, перспективно использование сетмелатонида, вещества, восстанавливающего нарушенную работу рецептора меланокортина (MC4R). Проведено многоцентровое рандомизированное 52-­недельное исследование этого препарата (ClinicalTrials.gov, NCT03746522), препарат обеспечил хорошее снижение массы тела у детей с синдромом Барде–Бидля, но при синдроме Альстрема эффективность препарата не удалось доказать [12].

Важность лечения ожирения, в том числе не только распространенного экзогенно-конституционального, но и орфанных, моногенных форм, трудно переоценить, именно поэтому важной инновацией в изменении структуры эндокринологической службы в стране стало создание особого Центра лечения ожирения на базе ГНЦ ФГБУ «НМИЦ эндокринологии» Минздрава России.

Любопытны и опубликованные в 2022 г. работы, потенциально способные изменить лечение классических форм врожденной дисфункции коры надпочечников (ВДКН), при которой соблюдение баланса между подавлением продукции андрогенов, по сути достижимым только на супрафизиологических дозах глюкостероидов, и отсутствием передозировки стероидов далеко не всегда возможно, особенно у взрослых. К настоящему времени несколько молекул доказали потенциальную эффективность — это Tildacerfont, предложенный для лечения взрослых [11]. По данным K. Sarafoglou и соавт., этот нестероидный антагонист рецептора 1-го типа к кортиколиберину обладает высокой связывающей активностью и активно снижает секрецию АКТГ, что позволяет у лиц с ВДКН снизить продукцию предшественников надпочечниковых андрогенов. Это уже вторая попытка выпустить подобные молекулы, и на основании двух законченных исследований (NCTJ03257464 и NCT03687242) получены данные о снижении продукции АКТГ, андростендиона и 17-оксипрогестерона при хорошей переносимости препарата. Надо сказать, что вопросы оптимальной биодоступности этого липофильного препарата, как и оптимальных дозировок, станут предметом дальнейших исследований.

Еще один аналогичный препарат, crinecerfont, находится во второй фазе изучения (NCT03525886), и данные по нему, недавно представленные группой авторов под руководством R. Auchus [13], показали, что этот пероральный нестероидный антагонист рецептора 1-го типа для кортиколиберина позволил уменьшить избыточное использование глюкокортикостероидов у взрослых лиц с ВДКН, при этом выявлялось дозозависимое снижение aндростендиона.

Разработан препарат Macimorelin — синтетический агонист рецептора к грелину для тестов на секрецию соматотропного гормона, прирост оценивается при пероральном (!) приеме [12]. Возможно, мы простимся с инсулиновой гипогликемией как тестом в поисках дефицита гормона роста? Впрочем, стоимость этого препарата сегодня так велика, что наша радость по поводу его появления может быть преждевременной.

Лечение ахондроплазии не входит в функцию эндокринолога в нашей стране, тем не менее многим врачам будет интересно сообщение, что FDA разрешило препарат Vosoritide (Voxzogo), вводимый ежедневно в виде инъекций, для лечения детей с этим заболеванием в возрасте от 5 лет с открытыми зонами роста. Генетически детерминированное заболевание, ахондроплазия, связано с аномалиями энхондрального окостенения, блокатором которого является СNP, натрийуретический пептид. По химической структуре Vosoritide является аналогом CNP, и его применение позволяет улучшить ростовой прогноз [14].

## В ОБЛАСТИ ЛЕЧЕНИЯ НАСЛЕДСТВЕННЫХ ДИСЛИПИДЕМИЙ

Нарушения обмена липопротеинов и дислипидемии относятся к эндокринным заболеваниям и в МКБ-10 кодируются шифром E78.

В 2022 г. в России зарегистрирован инновационный инъекционный препарат для лечения дислипидемии — инклисиран (торговое наименование Симбрава). Это первый и единственный в классе гиполипидемических средств препарат, созданный на основе малой интерферирующей РНК. Инклисиран вызывает расщепление матричной РНК и соответствующее снижение синтеза белка PCSK9 — пропротеиновой конвертазы субтилизин-кексинового типа 9. Этот белок «повинен» в уменьшении количества рецепторов холестерина липопротеинов низкой плотности (ХЛНП), а значит, в его сниженном захвате клетками и увеличении его концентрации в крови. Соответственно, угнетение синтеза белка PCSK9, напротив, способствует сохранению рецепторов ХЛНП на поверхности клеток, активному захвату холестерина из кровотока и снижению его концентрации в крови. Препарат инклисиран показан для лечения пациентов с первичной гиперхолестеринемией (семейной гетерозиготной и несемейной гиперхолестеринемией) или смешанной дислипидемией в качестве дополнения к диете [15]. Основанием для регистрации инклисирана стали результаты клинических испытаний III фазы ORION-9, -10 и -11 [16]. В клинических исследованиях приняли участие более 3,5 тысячи пациентов с сердечно-сосудистыми заболеваниями или гетерозиготной семейной гиперхолестеринемией, принимающих максимальные дозы статинов.

Препарат инклисиран в режиме 1 инъекция в 6 мес достоверно снижал уровень ХЛНП на 52% относительно исходного уровня к 17-му месяцу терапии и удерживал его снижение на 54% на постоянном уровне в течение последующего периода лечения.

Хочется верить, что 2023 г. станет годом внедрения наиболее эффективных методов диагностики и лечения, полученных в 2022 г., и годом новых успешных открытий.
